# Prognostic Value of Semi-Quantitative Metabolic Parameters on [18F]FDG PET/CT in Patients with Diffuse Large B-Cell Lymphoma at Diagnosis

**DOI:** 10.3390/curroncol33070392

**Published:** 2026-07-01

**Authors:** Emanuele Cencini, Federica Orsini, Marta Franceschini, Sara Fredducci, Mattia Bello, Emanuele Pacini, Marcello Bradaschia, Anna Sicuranza, Chiara Carrara, Paolo Bertelli, Monica Bocchia, Alberto Fabbri

**Affiliations:** 1 Unit of Hematology, Azienda Ospedaliera Universitaria Senese, University of Siena, 53100 Siena, Italy; m.franceschini@student.unisi.it (M.F.); sara.fredducci@student.unisi.it (S.F.); m.bello4@student.unisi.it (M.B.); emanuele.pacini@student.unisi.it (E.P.); m.bradaschia@student.unisi.it (M.B.); sicuranza4@unisi.it (A.S.); chiara.carrara@student.unisi.it (C.C.); bocchia@unisi.it (M.B.); 2 Unit of Nuclear Medicine, Azienda Ospedaliera Universitaria Senese, 53100 Siena, Italy; federica.orsini@ao-siena.toscana.it (F.O.); p.bertelli@ao-siena.toscana.it (P.B.)

**Keywords:** diffuse large B-cell lymphoma, positron emission tomography/computed tomography, treatment, prognosis

## Abstract

After first-line therapy with rituximab, cyclophosphamide, doxorubicin, vincristine, and prednisone (R-CHOP), 20–30% of patients with diffuse large B-cell lymphoma (DLBCL) have relapsed or refractory disease. Semi-quantitative volume parameters on [18F]Fluorodeoxyglucose positron emission tomography/computed tomography ([18F]FDG PET/CT), performed at diagnosis, could represent variables with prognostic influence. We retrospectively analyzed 53 consecutive patients, treated between 2016 and 2022. Semi-quantitative metabolic parameters, assessed by the software LIFEx, included total metabolic tumor volume (TMTV), total lesion glycolysis (TLG), Dmax and DmaxVox. All cases received R-CHOP/CHOP-like regimens with curative intent. The International Metabolic Prognostic Index (IMPI) was low in 43/53 cases (81.1%). CR was achieved in 49/53 patients (92.4%); 7/53 (13.2%) relapsed after achieving a CR. For the entire cohort, 2-year PFS and OS were 84.9% and 90.6%, respectively, while 5-year PFS and OS were 65.5% and 77.6%, respectively. IPI score, B symptoms, TMTV, Dmax and DmaxVox were associated with reduced PFS in an exploratory univariate analysis. IPI score was the only variable for which we found a significant association with reduced OS. In this exploratory analysis in a small, event-limited population, we suggest the baseline, semi-quantitative metabolic parameters of PET/CT at diagnosis could contribute to defining tumor burden and to predict PFS for DLBCL patients.

## Simple Summary

Diffuse large B-cell lymphoma (DLBCL) represents the most common subtype of non-Hodgkin lymphoma (NHL). After first-line therapy with rituximab, cyclophosphamide, doxorubicin, vincristine, and prednisone (R-CHOP), 20–30% of patients have relapsed or refractory disease. Recent studies showed that semi-quantitative volume parameters on pre-treatment [18F]Fluorodeoxyglucose positron emission tomography/computed tomography could represent variables with prognostic influence. We investigated, in a real-life population, the possible association between DLBCL outcome and total metabolic tumor volume (TMTV), total lesion glycolysis (TLG), Dmax and DmaxVox. In our study, TMTV, Dmax and DmaxVox demonstrated a significant correlation with reduced PFS in an exploratory univariate analysis only. IPI score was the only variable associated with reduced OS. According to the findings of our exploratory analysis in a small, event-limited population, we suggest these semi-quantitative metabolic parameters of PET/CT at diagnosis may contribute to future integrative prognostic models for DLBCL after further validation.

## 1. Introduction

Diffuse large B-cell lymphoma (DLBCL) represents the most common subtype of non-Hodgkin lymphoma (NHL) and accounts for about 30% of all NHL cases [1,2,3]. DLBCL is a heterogeneous lymphoid neoplasm with multiple morphologic variants, histologic subtypes, and genetic abnormalities, and it is characterized by an aggressive behavior [3,4]. After first-line therapy with rituximab, cyclophosphamide, doxorubicin, vincristine, and prednisone (R-CHOP), 20–30% of patients have relapsed or refractory (R/R) disease and overall prognosis is often poor, especially for patients who experience primary refractory DLBCL [5,6]. The therapeutic regimen polatuzumab-rituximab-CHP (pola-R-CHP) demonstrated an increased progression-free survival (PFS) at 2 years compared to R-CHOP in a phase III study. In addition, the greatest PFS benefit was reported for International Prognostic Index (IPI) 3–5 patients, even if overall survival (OS) did not differ significantly [7].

The identification of a high-risk DLBCL population is challenging, as the IPI score includes only clinical variables and it does not consider the biological heterogeneity of the disease [8]. Cell-of-origin (COO), based on gene expression profiling, is relevant, dividing patients into germinal center B-cell-like (GCB, better prognosis) and activated B-cell-like (poor prognosis), but it is not always available and the use of immunohistochemistry (IHC) has reproducibility issues [9,10].

The [18F]Fluorodeoxyglucose positron emission tomography/computed tomography ([18F]FDG PET/CT) represents a relevant imaging modality for the diagnosis, staging and response evaluation for patients with NHL, including DLBCL [11,12,13,14,15]. Semi-quantitative volume parameters on [18F]FDG PET/CT, performed at diagnosis, could represent variables with prognostic influence, regardless of the Deauville Score, which is currently used to perform response assessment after first-line therapy [16,17,18,19,20,21]. These parameters could act as indicators of the total disease burden which is metabolically active and include maximum standardized uptake value (SUVmax), total metabolic tumor volume (TMTV) and total lesion glycolysis (TLG) [22,23,24,25]. The TMTV was defined as the sum of the volumes of all hyper-metabolic lymphoma lesions, while TLG was defined as the product of the mean SUV (SUVmean) and the TMTV for each lesion [22,23,24,25]. SUVmax was determined at the site of highest uptake, and it reflects the metabolic intensity of the most active neoplastic cells [22,23,24,25]. Unfortunately, image algorithms and PET/CT devices could significantly affect its evaluation [22,23,24,25]. Consequently, TMTV and TLG could have greater accuracy for the prognostic evaluation of DLBCL, and high TMTV and TLG were associated with reduced PFS and OS [16,17,18,19,20,21]. The threshold for the TMTV was determined with several different methods, including a cut-off value of 2.5 or 4 for the SUV and a SUV value > 41% of the maximum SUV of each single lesion [26]. However, there is currently no gold standard for the best SUVmax ratio for measuring the TMTV, and the heterogeneity of lesion distribution is not adequately considered with TMTV evaluation [22,23,24,25,26].

Indeed, the distance between the two lesions that are the furthest apart (Dmax), a PET feature reflecting disease dissemination, was introduced and demonstrated to be effective at stratifying DLBCL cases, regardless of IPI score [27]. Furthermore, a new prognostic model, the International Metabolic Prognostic Index (IMPI), was built, including TMTV, age and stage [28,29,30]. According to this background, we aimed to investigate the prognostic impact of these metabolic parameters, independently or in combination with clinical variables, in a real-life population of patients with DLBCL receiving first-line therapy.

## 2. Materials and Methods

### 2.1. Study Population

In this observational, single-center, real-world study, we retrospectively enrolled 53 consecutive, newly diagnosed DLBCL patients treated from December 2016 to December 2022. Diagnosis was made according to the 2016 World Health Organization (WHO) Classification of Tumours of Haematopoietic and Lymphoid Tissues [31]. For IHC analysis, we used a cut-off of 40% and 50% for MYC and BCL2, respectively. To identify double-hit/high-grade B-cell lymphoma, FISH for MYC rearrangement was performed on patients with MYC > 40% in IHC or Ki67 proliferation index ≥ 80%. If positive, subsequent FISH for BCL2 and BCL6 rearrangements was performed [1,31]. All patients were managed at the Hematology Unit of the Azienda Ospedaliera Universitaria Senese, according to everyday clinical practice within the National Health Service. We enrolled all DLBCL patients treated with curative intent, regardless of histopathology. At our institution, the preferred first-line therapy was R-CHOP; dose-adjusted etoposide, prednisone, vincristine, cyclophosphamide, doxorubicin, and rituximab (R-DA-EPOCH) was considered for double-expressor DLBCL. Moreover, we enrolled patients who had undergone a PET/CT scan with [18F]FDG at diagnosis, before receiving treatment, who had an evaluable treatment response and for whom survival data were available up to the last visit. All PET/CT investigations were performed and analyzed by the experienced medical staff of the Nuclear Medicine Unit. Two nuclear physicians were involved in the study and conjointly reviewed [18F]FDG PET/CT-based images. PET readers were blinded to clinical outcomes. PET/CT analysis was conducted according to European Association of Nuclear Medicine procedural guidelines.

The study was approved in accordance with Institutional Review Board requirements and the Declaration of Helsinki and its amendments (Comitato Etico Regionale per la Sperimentazione Clinica della Regione Toscana Sezione: AREA VASTA SUD EST, protocol approval on 17 November 2025, protocol code SI_PET_NHL, protocol number 29880). Since it is a retrospective study, patients had already received diagnosis and treatment at the time of study approval. Each living patient signed an informed consent form at the first available visit after the approval date. Regarding the participants who died, consent was considered to have been acquired by Italian law, according to Authorization n.9/2016—General Authorization to Process Personal Data for Scientific Research (date of authorization was 15 December 2016). The medical records were reviewed to calculate IPI and IMPI scores. Baseline patient information, the treatment lines administered and survival data were collected in an electronic database.

Clinical data were collected regarding both first-line therapy and subsequent regimens for patients with R/R disease. Quantitative and semi-quantitative metabolic parameters derived from PET/CT analysis at diagnosis, including TMTV, TLG, Dmax, and DmaxVox (the distance between the two outermost voxels) were also calculated. Individual prognostic variables and the IPI and IMPI scores, derived respectively from clinical data and from both clinical and metabolic data of the patients, were included in the survival analyses.

Response to treatment and survival were reported according to intention-to-treat. We identified PFS as the primary endpoint, while OS, complete response (CR) rate, second-line regimens, causes of death and the identification of variables that could influence disease prognosis were secondary endpoints.

### 2.2. PET/CT Acquisition and Analysis

All images were obtained with a PET/CT scanner (Ingenuity TF PHILIPS, Cleveland, OH, USA), for which regular daily, monthly, and annual quality checks were performed by the Medical Radiology Technical staff of the Nuclear Medicine Unit and the Health Physics Unit of our institution. All patients underwent the PET/CT examination within two weeks of starting therapy. All patients had fasted for at least 6 h, and their blood glucose levels were below 160 mg/dL. The total administered radiotracer was 3.5 MBq/kg (minimum 185 MBq). The injection was performed using an automatic injector, following the establishment of venous access with a 22-gauge cannula. After the injection, each patient was kept resting in a dedicated chair for approximately 50–60 min.

PET images were acquired for one minute and thirty seconds per bed position for a total of about 9–10 bed positions, depending on the patient’s height, with the subject lying supine at free breathing. PET reconstruction was performed using the iterative BLOB-OS-TF method (Subset 33, three iterations, Gaussian post-filter with 4 mm FWHM). The CT image acquisition process was a low-dose scan at tube voltage 120 kV, with automatic specific calculation varying from 50 to 100 milliampere-seconds (mAs) of tube current and a slice thickness of 5 mm. The low-dose CT scan was used for attenuation-correction reconstruction of PET images and for the precise localization of the uptake foci.

The whole-body PET image sequences (from the skull base to the middle of the femur) in anonymized Digital Imaging and Communications in Medicine format were imported into LIFEx (www.lifexsoft.org accessed on 2 February 2026) software v7.6 for the automatic calculation of SUVmax, TMTV, TLG, Dmax and DmaxVox parameters [32]. The volumes of interest (VOIs) were delineated semi-automatically including the axial, sagittal, and coronal planes. Physiological uptake VOIs (mainly brain, kidney and excretory pathways) were manually excluded from the automatic calculation performed by the software. The segmentation method included lesions with a SUV ≥ 4 as the threshold for delineating lymphoma lesions for TMTV calculation; this approach was preferred to increase specificity for tumor and interobserver reproducibility [33]. In addition, included lesions had a volume of at least 3 cm^3^. IMPI score was calculated as previously published [28].

### 2.3. Treatment Regimen and Response Assessment

All patients received chemoimmunotherapy with curative intent (R-CHOP or CHOP-like, R-DA-EPOCH) for four–six cycles, followed by radiation therapy (RT) as consolidation for localized disease. The rituximab-polatuzumab-CHP therapeutic regimen obtained reimbursement in Italy under the National Health System on 20 December 2023. Therefore, this regimen was not administered to our patients. R-CHOP was given every 21 days at full dose (rituximab 375 mg/m^2^ intravenously on day 1, cyclophosphamide 750 mg/m^2^, doxorubicin 50 mg/m^2^, vincristine 1.4 mg/m^2^ with a maximum dose of 2 mg on day 2, prednisone 40 mg/m^2^ on days 2–6). Doxorubicin was substituted by a non-pegylated liposomal formulation (R-COMP) for patients with cardiac comorbidities or high cardiovascular risk [34]. End-of-treatment high-dose methotrexate (two cycles of 3 g/m^2^) was administered as CNS prophylaxis to patients presenting with CNS-IPI score 4–6 and multiple extranodal sites, including renal, breast or testicular involvement. In addition, patients who had been deemed ineligible for high-dose methotrexate received CNS prophylaxis with four doses of intrathecal methotrexate (12 mg). 

Patients received prophylaxis against P. jirovecii with trimethoprim-sulfamethoxazole 160/800 mg twice a day for 2 days a week. HBV-positive patients received lamivudine as prophylaxis until 12 months after treatment, while HCV carriers were considered to receive direct-acting antiviral therapy, as previously published [35]. Patients who achieved a CR were defined as responders, while patients with partial response (PR), stable disease (SD) or progressive disease (PD) were defined as non-responders. Treatment response after the completion of therapy was determined according to the 2014 Lugano classification [36]. All patients were followed up through periodic clinical and radiological monitoring.

Both hematological and extrahematological toxicity were assessed at each cycle using the Common Terminology Criteria for Adverse Events (CTCAE) system v5.0.

### 2.4. Statistical Analysis

Patients’ characteristics were analyzed according to descriptive statistics. Qualitative variables were described using absolute frequencies and percentage relative frequencies. Quantitative variables were summarized in terms of median and range. The Mann–Whitney U test was used to determine if there was a significant difference between the median values of two groups. For TMTV, both the best cut-off for PFS determined by receiver operating characteristic (ROC) curve and the median value were calculated. We acknowledge that the Area Under Curve (AUC), often referred to as the area under the ROC curve, measures the effectiveness of a binary classification model and its range varies from 0.5 to 1.0 for predictive models (although mathematically the theoretical range is from 0 to 1). The practical interpretation of the AUC is that a value between 0.5 and 0.7 indicates the model is inaccurate, even if slightly better than chance. Therefore, we considered median-based analysis as the primary analysis and the ROC-derived cut-off analysis as exploratory.

Other metabolic parameters related to PET/CT were analyzed dichotomously based on the median value to determine their correlation with survival. OS was calculated as the time from the 1st day of treatment until death from any cause, or the last follow-up for censored cases. PFS was calculated as the time from the 1st day of treatment until disease progression, relapse, death from any cause or the last follow-up for censored cases. Survival curves were assessed using Kaplan and Meier plots and log-rank test for significant associations; a *p* value < 0.05 was interpreted as statistically significant. We used a Cox proportional hazards model to evaluate significance of association between investigated covariates and survival for the multivariate analysis. Specifically, univariate analysis was first performed, and factors with *p* < 0.05 were selected for multivariate Cox proportional hazards regression to identify independent predictors of survival outcome. We included TMTV > median in the set of covariates and we excluded TMTV > ROC-derived cut-off to avoid potential collinearity. Similarly, we excluded the components of IPI score to avoid an overlap between a composite score and its constituent variables. Our findings are reported as a hazard ratio (HR), with a 95% confidence interval (CI).

All statistical analyses were performed with Statistical Software MedCalc, 19.6 (MedCalc Software Ltd., Ostend, Belgium; https://www.medcalc.org; accessed on 3 March 2026).

Being a retrospective study, there was no predetermined statistical sample size, but consecutive patients from 2016 to 2022 were analyzed. We chose the time frame to identify patients diagnosed and treated in a homogeneous way and to ensure a follow-up of at least 2 years from the start of therapy for all patients, which represents a key cut-off for assessing long-term survival in DLBCL.

## 3. Results

### 3.1. Characteristics of Patients

The clinical characteristics of the whole cohort are represented in Table 1. All patients received a physical examination, complete blood cell count, PET/CT scan and bone marrow biopsy at diagnosis. Out of 54 patients who underwent PET/CT scans, one case was discarded as it could not be evaluated within the LIFEx software for technical aspects. Out of 53 enrolled patients, the median age at diagnosis was 66 years (range 34–88 years), with a slight female predominance (27/53 patients, 50.9%). ECOG Performance Status was 0–1 in 50/53 patients (94.3%); 12/53 (22.6%) and 14/53 (26.4%) patients presented with B symptoms and elevated lactate dehydrogenase (LDH), respectively. The population was distributed fairly evenly between localized and advanced stages; 26/53 (49.1%) and 27/53 (50.9%) patients presented with stage I-II and stage III-IV disease, respectively. With regard to histopathology, one patient met diagnostic criteria for primary cutaneous DLBCL, leg type. In addition, 11/53 patients (20.7%) presented with double-expressor DLBCL. COO was determined by IHC using the Hans algorithm; 22/53 (41.5%) and 31/53 (58.5%) patients were GCB- and non-GCB-type, respectively. IPI score was 3–5 for 32.1% of total cases (17/53). Central nervous system (CNS)-IPI score was low (0–1), intermediate (2–3) or high (4–6) for 17/53 (32.1%), 31/53 (58.5%) and 5/53 (9.4%) patients. Interestingly, IMPI score was low in 43/53 cases (81.1%) and only one case had high-risk disease.

### 3.2. Baseline PET Metabolic Parameters

The descriptive statistics of baseline PET features are reported in Table 2. Patients without measurable disease on baseline PET/CT scan (*n* = 4) before treatment were considered to have a TMTV = 0 mL. This subgroup included two cases with absence of macroscopic disease after surgical diagnostic biopsy and two cases with follicular large B-cell lymphoma and single lesions with a SUV mean under the identified threshold for TMTV calculation. For patients with TMTV = 0 or no measurable disease, DmaxVox was assigned a value of 0. For patients with stage IA disease and a single area of uptake (8/53, 15.1%), Dmax was not evaluable.

The median SUVmax, TMTV, TLG, Dmax and DmaxVox were 16.45 (range 0–47.1), 224.3 mL (range 0–5414.6), 1263.3 g (range 0–36,802), 15.7 cm (range 0–93) and 22.5 cm (range 0–104.6), respectively. The best cut-off for TMTV according to the ROC curve was 216.6 mL (AUC was 0.551).

### 3.3. Treatment Response

First-line regimens were administered with curative intent and included R-CHOP (31/53 patients, 58.5%), R-COMP (17/53 patients, 32.1%) and R-DA-EPOCH (5/53 patients, 9.4%). All patients who received R-DA-EPOCH were younger (<65 years old) and presenting with high tumor burden, double-expressor DLBCL. Overall, six cycles of chemoimmunotherapy were planned for 36/53 patients (67.9%), while the remaining 17 patients (32.1%), who had stage I-II disease, received an abbreviated regimen with no more than four treatment cycles and RT as consolidation. The treatment schedule planned at diagnosis was prematurely discontinued in 3/53 patients (5.7%), due to PD (one case after R-CHOP) or unacceptable toxicity (two cases). The treatment that conditioned the unacceptable toxicity was R-CHOP and R-DA-EPOCH (one case each). End-of-treatment high-dose methotrexate was administered as CNS prophylaxis to two patients, who had CNS-IPI score 4–6 and multiple extranodal sites, including renal, breast or testicular involvement. In addition, three elderly patients who had been deemed ineligible for high-dose methotrexate received four doses of intrathecal methotrexate (12 mg).

Overall, 49/53 patients (92.4%) achieved a CR and 3/53 patients (5.7%) a PR, while the remaining case (1.9%) experienced SD/PD (this patient received R-DA-EPOCH but an appropriate dose intensity was not maintained due to hematological toxicity). Regarding patients in PR, one patient died due to septic shock before performing the fifth cycle of R-CHOP, one patient received second-line therapy and the remaining case, with a single site of disease persistence at PET/CT scan performed at the end of induction, received RT as consolidation and converted to CR.

In the entire cohort, 9/53 patients (17%) received second-line therapy, including the above-mentioned two patients who had an unsatisfactory response to first-line therapy and 7/53 patients (13.2%) who relapsed after achieving a CR.

Second-line regimens included rituximab, oxaliplatin, high-dose cytarabine and dexamethasone (R-DHAOx, 3/53 cases, 5.7%), rituximab, gemcitabine and oxaliplatin (R-GemOx), rituximab, cyclophosphamide, mitoxantrone, vincristine, etoposide, bleomycin and prednisone (R-VNCOP-B), tafasitamab plus lenalidomide, rituximab and bendamustine (due to disease relapse as follicular lymphoma), RT (due to an isolated, cutaneous relapse) and low-dose steroids as palliative care (one case for each regimen). Only 4/9 patients achieved a response (CR or PR) after second-line therapy; remarkably, all patients who received R-DHAOx achieved a SD/PD and underwent chimeric antigen receptor (CAR)-T cell therapy as a third-line regimen (CAR-T not yet approved as second-line regimen).

### 3.4. Survival Analysis

**Figure 1:**
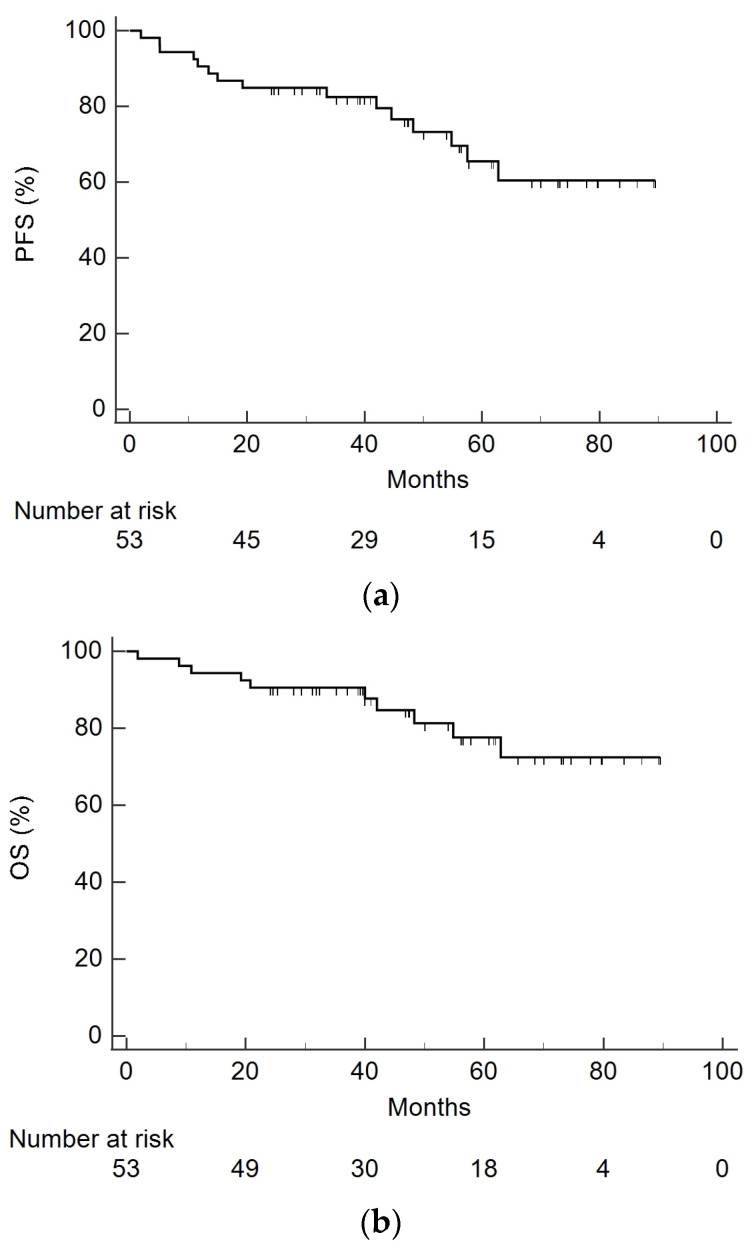
Progression-free survival (**a**) and overall survival (**b**) for the entire cohort.

For the entire cohort, after a median follow-up of 47.3 months, the 2-year PFS and OS were 84.9% (95%CI 76–95%) and 90.6% (95%CI 82–98%), respectively, while the 5-year PFS and OS were 65.5% (95%CI 50–82%) and 77.6% (95%CI 64–91%), respectively (Figure 1a,b).

**Figure 2:**
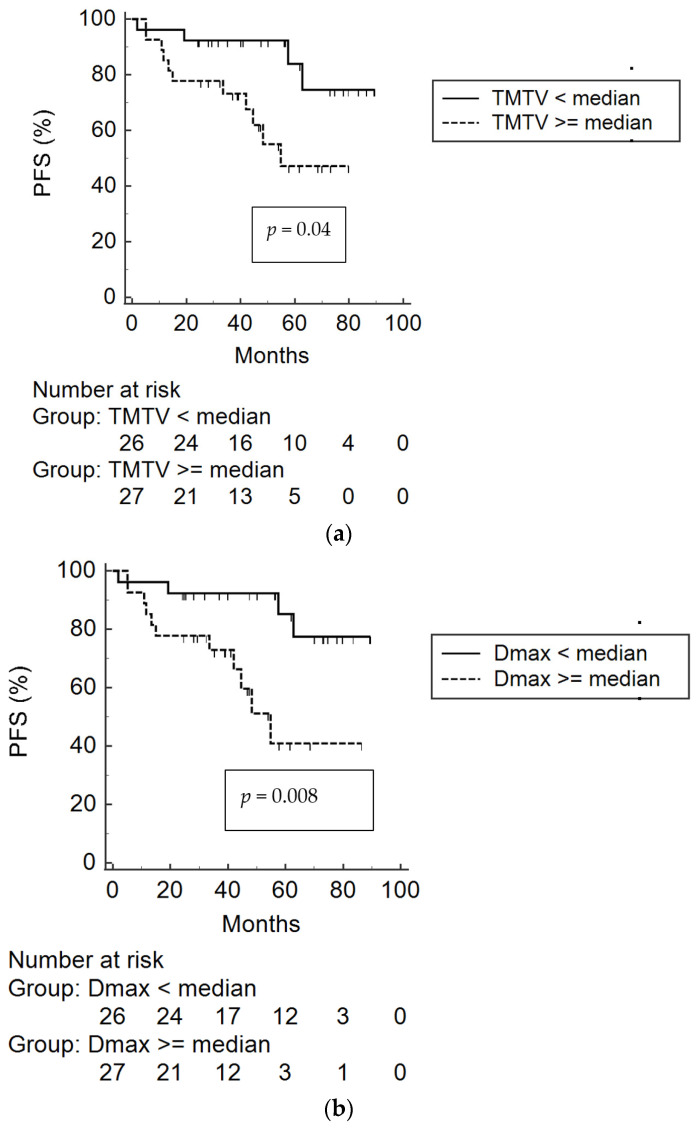

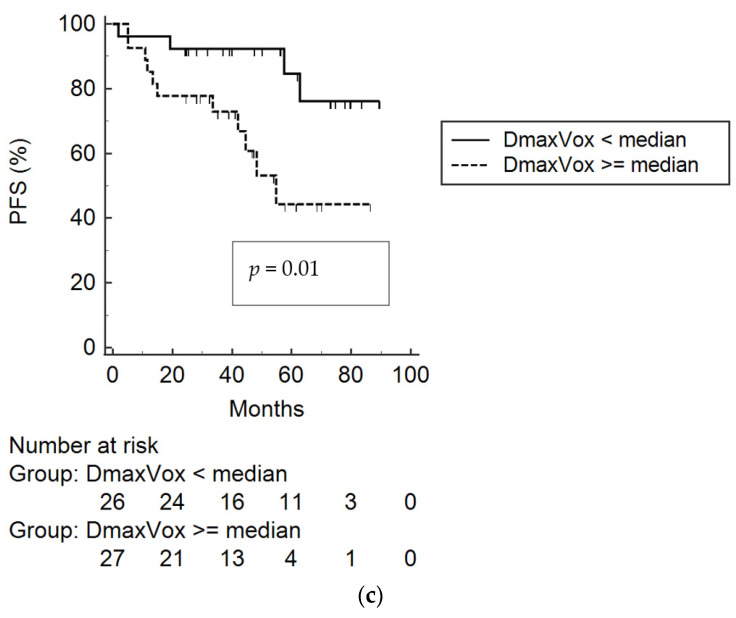
Progression-free survival according to TMTV (**a**), Dmax (**b**) and DmaxVox (**c**). The reported *p* values are log-rank *p* values.

Overall, we observed 15 PFS events and 10 OS events. The results of the univariate analysis revealed a significant correlation between high TMTV (higher than the median value), Dmax, DmaxVox and reduced PFS (Figure 2a–c). As an exploratory analysis, high TMTV with best cut-off value assessed by ROC curve was also associated with reduced PFS (Table 3). Furthermore, the 2-year PFS for patients with high versus low TMTV was 77.8% vs. 92.3%. Lastly, we tried to perform separate sensitivity analyses for TMTV > median after excluding the four patients with TMTV = 0 and for Dmax > median after excluding the eight patients with Dmax = 0, as reported in Table S1. In this univariate analysis, a significant correlation between TMTV and Dmax higher than the median value and reduced PFS was maintained.

**Figure 3:**
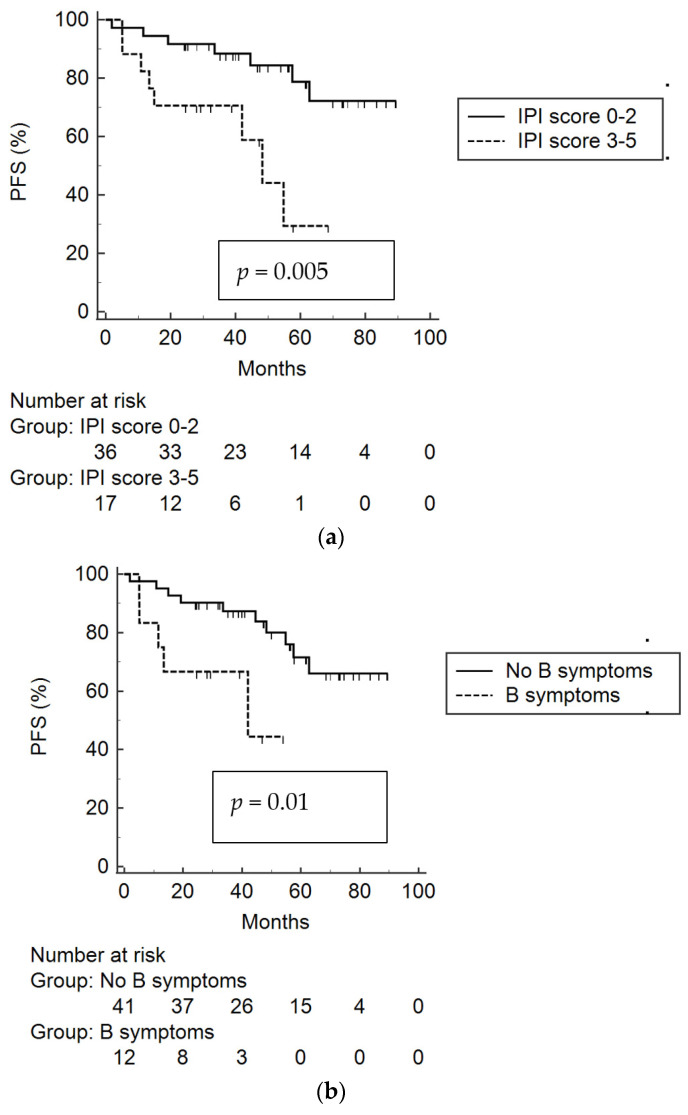
Progression-free survival according to IPI score (**a**) and B symptoms (**b**). The reported *p* values are log-rank *p* values.

Clinical factors such as IPI score 3–5 and B symptoms were also correlated with inferior PFS (Figure 3a,b). Table 3 represents the HR with 95% CI for PFS for each clinical and PET-derived variable. In the multivariate Cox proportional hazards model, we included in the set of covariates B symptoms, IPI score 3–5, TMTV > median, Dmax > median, and DmaxVox > median, but none of these variables was confirmed as an independent predictor of PFS.

**Figure 4:**
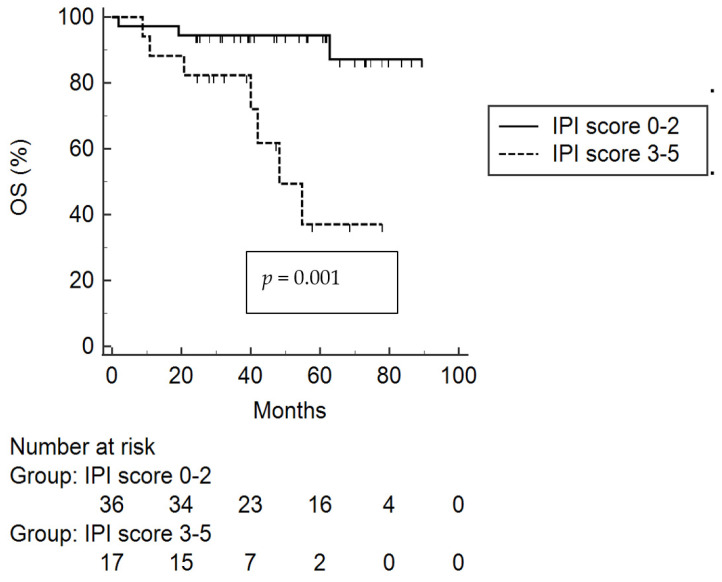
Overall survival according to IPI score. The reported *p* values are log-rank *p* values.

At the last follow-up, 43/53 patients were alive; causes of death included PD (four cases), death of unknown origin while in CR (three patients, aged > 80 years), infectious complications (two cases), and secondary acute myeloid leukemia (one case). IPI score was the only variable for which we found a significant association with reduced OS (Table 4 and Figure 4).

Lastly, we tried to perform separate sensitivity analyses for TMTV > median after excluding the four patients with TMTV = 0 and for Dmax > median after excluding the eight patients with Dmax = 0, as reported in Table S2. In this univariate analysis, we confirmed a lack of significant correlation between TMTV and Dmax higher than the median value and reduced OS.

We tried to exclude four patients with TMTV = 0 and eight patients with Dmax = 0 with comparable results (Table S2). Since only IPI score was significant in the univariate OS analysis and the number of OS events was small, we have not constructed an OS multivariate model.

Remarkably, SUVmax and IMPI score were not correlated with either PFS or OS. Finally, we have compared, according to the metabolic parameters evaluated in the study, the two groups with low IMPI score vs. intermediate and high IMPI score. As expected of the construction, due to TMTV itself being a component of IMPI score, we observed a higher median TMTV in the intermediate/high-IMPI group. Consequently, it does not represent an independent finding, and no other significant differences emerged, as illustrated in Table 5.

## 4. Discussion

This analysis supports the possibility that baseline semi-quantitative PET parameters could have prognostic relevance for PFS in DLBCL patients receiving first-line R-CHOP or R-CHOP-like regimens [16,17,18,19,20,21]. Specifically, elevated TMTV, Dmax and DmaxVox were associated with reduced PFS, while TLG did not demonstrate any prognostic relevance. [18F]FDG PET/CT demonstrated a fundamental and consolidated role in the staging and response assessment of DLBCL [11,12,13,36]. Standard prognostic assessment for DLBCL includes IPI score and its successors, such as revised IPI and NCCN-IPI [4,37,38]. Among DLBCL patients with intermediate- or high-risk IPI scores in the POLARIX study, the risk of progression or death was inferior among those who received pola-R-CHP than among those treated with R-CHOP [7]. In the last years, considering the crucial prognostic role of PET-CT scan and its semi-quantitative parameters at baseline in Hodgkin lymphoma, these variables emerged as potential survival predictors for DLBCL as well [13].

ROC analysis identified 216.6 mL as the optimal cut-off for TMTV, being close to the median value for our cohort and consistent with values reported in previously published studies, even if somewhat different to values of 300–396 mL observed in other series [18,19,39,40,41,42,43,44,45]. Interestingly, EHA guidelines suggested a value of 220 mL for TMTV as the bulk definition [46]. TMTV represents the volume of all pixels recorded on PET images that exceed a predetermined SUV threshold. This discrepancy could be mainly due to differences in the methods used to determine the optimal cut-off for including lesions in the TMTV determination [26,45]. These differences could limit the standardization of TMTV evaluation and the reproducibility of results. However, many retrospective studies showed comparable findings and confirmed the potential predictive power of TMTV for survival, regardless of Ann Arbor stage and IPI score [18,19,39,40,41,42,43,44]. A significant proportion of studies used an absolute threshold of 2.5 to define TMTV, while a limited number used the 41% of maximal SUV for TMTV calculation [39,40,41,42,43,44,45]. Recently, an international working group was established, that indicated the best cut-off value as SUV > 4 [26]. Our study, although conducted with a limited number of patients, suggests a possible prognostic role for PFS of baseline TMTV using an SUV > 4 threshold, in accordance with the LYSA study by Malmon and colleagues [26].

In our study, the SUVmax value at diagnosis did not significantly correlate with survival. This could be due to the small sample size, but also to the need to use this parameter as a dynamic variable. The limited prognostic value of baseline SUVmax could be due to the fact that DLBCL is an aggressive disease, in which prognosis is not related to the [18F]FDG PET scan avidity recorded in the most metabolically active lesion, but mainly to the overall burden of metabolically active disease [47]. Casasnovas and colleagues demonstrated that a reduction in SUVmax value during treatment had a prognostic influence, rather than baseline assessment [48]. In a larger study, in which SUVmax was extensively investigated in a training cohort and in a validation cohort, it did not demonstrate a significant correlation with PFS and OS [48].

Moreover, TLG, a combination parameter derived by multiplying TMTV by SUV mean, did not demonstrate a significant correlation with PFS and OS. Available data showed conflicting results on TLG prognostic value [14,19,49,50]. The retrospective analysis of the phase III GOYA study showed that the optimal cut-off of 3004 g for TLG was associated with inferior PFS [19]. However, in the study by Islam and colleagues, elevated TLG was not associated with reduced survival [14]. According to these findings, TLG could represent a less robust parameter than TMTV for DLBCL patients. In addition, some studies reported neither TMTV nor TLG as prognostic predictors for PFS and OS [51].

Unlike TMTV, TLG and SUVmax, which do not account for the spatial lesion distribution, limited available data exists regarding the use of dissemination parameters, such as Dmax and DmaxVox, as prognostic factors for DLBCL patients [15,20,21,27]. Interestingly, in our population, elevated Dmax and DmaxVox were associated with reduced PFS in univariate analysis and a trend towards OS. Dmax represents the distance between the two lesions that are the furthest apart and it is easy to calculate, unlike other sophisticated radiomic features [27]. Dmax and TMTV showed an independent prognostic value, further suggesting they could define two different disease features, such as tumor burden and dissemination, respectively [21]. In a recently published study, a Dmax of 53.9 cm was associated with inferior PFS and included in a prediction nomogram, together with TMTV and clinical parameters [15]. However, the use of Dmax was limited to patients with advanced stage disease or, in any case, to those with more than one lesion [21,27]. In our study, there were eight patients with localized disease in which the second lesion was not analyzable by the software or had been removed by diagnostic lymph node biopsy performed before PET/CT scan. These cases were included in the analysis in the group with Dmax below the median by assigning them the value of 0. We acknowledge that the exclusion of these patients could prevent the achievement of prognostic significance in a population with reduced sample size. Remarkably, we adopted the same methodological approach as Vergote and colleagues [29]. Regarding Dmax, Vergote and colleagues had a range of 0–128 cm and an interquartile range of 0–50 cm [29]. In addition, we tried to remove the four patients with TMTV = 0 and the eight patients with Dmax = 0, and we found no significant difference in survival analysis (Tables S1 and S2).

The DmaxVox, on the other hand, appears to be a more robust metabolic parameter as it represents the distance between the outermost voxels of the two most distant hypermetabolic sites and can be calculated in all patients, regardless of the number of lesions [20]. In such cases, it may reflect the spatial extent of an individual lesion rather than inter-lesional dissemination. In a recently published study using LIFEx software, an elevated DmaxVox value was associated with reduced survival, and DmaxVox was successfully combined with TMTV to better stratify DLBCL patients [20].

Regarding clinical parameters, the IPI is the most widely used score in clinical practice, and its prognostic value is well established [1,2,3,4]. The confirmation of statistical significance in our analysis could validate our cohort as potentially representative of the DLBCL patient population, despite the small sample size.

With the purpose of combining clinical parameters (age as an estimate of the patient’s biological reserves and stage as an index of disease dissemination) with metabolic parameters, particularly the TMTV, we applied the very recent IMPI score developed by Mikhaeel and colleagues to our cohort [28,29,30]. In this study, the prognostic model included TMTV (with SUV > 4, as in our study)-age-stage, identified three risk groups and demonstrated a higher predictive value than both the TMTV and the IPI [28]. Conversely, in the study by Vergote and colleagues, the IMPI score was validated as a predictive score for survival, but its superiority compared with the IPI score was not confirmed [29]. In our cohort, which was mainly represented by low-risk patients, the conclusion that IMPI was not correlated with survival should be interpreted cautiously and attributed to the small sample size and risk-group imbalance rather than a true absence of prognostic value. When compared to those of the studies from which the IMPI score was derived, in our population there was a lower number of patients with advanced stage disease (50.9%) when compared to the HOVON-84 (82.2%) and GSTT (68.2%) studies, but the percentage was similar to that of the SAKK (56.7%) and PETAL (58.3%) studies. In addition, the median TMTV (224.3 mL) in our population was lower when compared to that of the aforementioned merged studies (307.9 mL) [28]. We acknowledge that we applied the IMPI score to a cohort that was predominantly low-risk (81.1% low IMPI), making any conclusion about its performance in our study unreliable.

However, our findings were similar to those of Vergote and colleagues in terms of median TMTV (219 mL) and population distribution according to IMPI score. In this study, as in our analysis, IMPI was not able to replace IPI score as a prognostic tool for PFS and OS assessment [29]. Therefore, our study could be framed as an exploratory, single-center, real-world replication/validation analysis, rather than one introducing an innovative prognostic approach.

Overall, survival curves in our population generated results in line with the most recent survival data, with 65.5% and 77.6% of patients who were progression-free and alive at 5 years from diagnosis, respectively. The difference between PFS and OS could be due to the increasing availability of new therapies for those patients who failed the first-line regimen, such as CAR-T or bispecific antibodies. Our findings are consistent with previously published studies and a recent meta-analysis, in which TMTV was reported as a strong predictor of inferior PFS, as compared to SUVmax and TLG [22,23,24,25,26]. The main limitation of our study is represented by its retrospective and single-center nature. We acknowledge that the sample size of 53 patients is small, which limits statistical power, particularly for multivariate analysis. Specifically, the current results are exploratory and should be interpreted cautiously given the limited sample size, the small number of PFS and OS events and the lack of independent prognostic significance in multivariate analysis. For PFS analysis, we strongly restate the exploratory nature of multivariate analysis, as the number of covariates may be excessive relative to the number of events, increasing the risk of overfitting and unstable estimates, as reflected in the wide confidence intervals. Moreover, the proportional hazards assumption was not checked. In addition, our population was treated with different regimens; thus, our analysis could be underpowered to detect a statistical significance in multivariate analysis for metabolic parameters such as Dmax, TMTV, and DmaxVox. The limited number of analyzed cases derived both from the limited time period since the introduction of PET/CT scan at our institution and from technical limitations related to the analysis of images through dedicated software. Furthermore, due to an AUC between 0.5 and 0.7 having limited discriminatory power, we considered median-based analysis as the primary analysis and the ROC-derived cut-off analysis as exploratory. In our single-center study with retrospective design, due to the lack of an external validation cohort, the generalizability of findings is severely limited. We acknowledge the limitations of deriving and testing a cut-off for ROC curve in the same small cohort without external validation. Lastly, although most patients received R-CHOP or R-COMP, the cohort also included R-DA-EPOCH (selectively used in younger patients with high tumor burden and double-expressor disease) and abbreviated chemotherapy plus radiotherapy for localized disease. We acknowledge, as a limitation, that the study is not powered to assess the prognostic impact of therapeutic regimens.

On the other hand, the main strength and the relatively more distinctive aspect of our study are represented by the comprehensive analysis of a consecutive series of DLBCL patients treated with curative intent, of all the semi-quantitative PET parameters, including dissemination parameters such as Dmax and DmaxVox, and of the IMPI score.

In our opinion, TMTV at diagnosis should be combined with clinical variables with the aim to improve the prognostic value of IPI score and to define a subgroup of DLBCL patients with the highest risk of first-line treatment failure. In a pivotal study, TMTV provided additional stratification for DLBCL patients with IPI score 3–5, which could be further divided into two subgroups with different PFS and OS [19]. In the near future, prospective clinical trials could investigate an integrated prognostic model, in which metrics extracted from baseline PET could play a relevant role, together with COO, in circulating tumor DNA and tumor microenvironment [1,2,3,4,8,52]. In addition, for older patients, TMTV could be associated with the EPI score, with the aim of improving the identification of patients at high risk of early mortality [53].

## 5. Conclusions

In conclusion, in our study, baseline PET-derived TMTV, Dmax, and DmaxVox, together with IPI score and B symptoms, were associated with PFS in an exploratory univariate analysis only. None of them was confirmed as an independent predictor of PFS in multivariate Cox analysis, and for OS only IPI score was significant in this small, event-limited population who received immunochemotherapy. The standardization and optimization of these parameters could offer innovative insights into the assessment of disease burden for DLBCL patients. Moreover, these parameters may contribute to future integrative prognostic models after further validation. The upfront identification of DLBCL patients with the highest risk of treatment failure may lead to alternative first-line regimens and/or an early disease assessment, in the context of the growing availability of targeted second-line regimens, including CAR-T, rituximab-polatuzumab-bendamustine and tafasitamab plus lenalidomide. We hope that the continuous improvement of software technology can enable the rapid availability and elevated reproducibility of results for baseline semi-quantitative PET parameters in clinical daily practice.

## Figures and Tables

**Table 1:** Characteristics of patients at diagnosis.

Characteristic	Number of Patients (%)
Age: median [range]	66 [34–88]
Male	26/53 (49.1%)
ECOG PS 0–1	50/53 (94.3%)
B symptoms	12/53 (22.6%)
Elevated LDH	14/53 (26.4%)
Ann Arbor Stage I–II	26/53 (49.1%)
Ann Arbor Stage III–IV	27/53 (50.9%)
IPI score 0–2	36/53 (67.9%)
IPI score 3–5	17/53 (32.1%)
CNS-IPI score 0–1	17/53 (32.1%)
CNS-IPI score 2–3	31/53 (58.5%)
CNS-IPI score 4–6	5/53 (9.4%)
Histopathology	
Double expressor	11/53 (20.7%)
T-cell rich DLBCL	2/53 (3.8%)
Leg-type DLBCL	1/53 (1.9%)
Extranodal sites	8/53 (15.1%)
Bulky disease (≥7 cm)	9/53 (17%)
GCB-type	22/53 (41.5%)
Non-GCB-type	31/53 (58.5%)
IMPI score low	43/53 (81.1%)
IMPI score intermediate	9/53 (17%)
IMPI score high	1/53 (1.9%)
First-line treatment	
R-CHOP/COMP	48/53 (90.6%)
R-DA-EPOCH	5/53 (9.4%)

Abbreviations: PS, performance status; LDH, lactate dehydrogenase; IPI, international prognostic index; CNS, central nervous system; GCB, germinal center B-cell-like; IMPI, international metabolic prognostic index; R-CHOP, rituximab, cyclophosphamide, doxorubicin, vincristine, prednisone; R-COMP, doxorubicin was substituted by a non-pegylated liposomal formulation; R-DA-EPOCH, etoposide, prednisone, vincristine, cyclophosphamide, doxorubicin, and rituximab.

**Table 2:** Baseline metabolic parameters.

Variable	Mean	Median	Range
SUVmax	16.8	16.45	0–47.1
TMTV	633	224.3	0–5414.6
TLG	3747.8	1263.3	0–36,802
Dmax	29.13	15.7	0–93
DmaxVox	35.7	22.5	0–104.6

Abbreviations: SUVmax, maximum standardized uptake value; TMTV, total metabolic tumor volume; TLG, total lesion glycolysis; Dmax, the distance between the two lesions that are the furthest apart; DmaxVox, the distance between the two voxels that are the furthest apart.

**Table 3:** Predictive factors of progression-free survival.

Variable	Univariate Cox Analysis HR (95% CI)	*p* Value	Multivariate Cox Analysis HR (95% CI)	*p* Value
Age ≥ 65 years old	1.093 (0.379–3.149)	0.868	/	
Male	1.206 (0.436–3.332)	0.716	/	
Stage III-IV	2.398 (0.856–6.712)	0.096	/	
B symptoms	6.831 (1.442–32.348)	0.015	2.264 (0.621–8.249)	0.215
IPI score 3–5	5.688 (1.684–19.216)	0.005	2.018 (0.505–8.058)	0.32
ECOG ≥ 2	0.988 (0.131–7.454)	0.988	/	
LDH	2.211 (0.798–6.126)	0.127	/	
TMTV > median	2.933 (1.050–8.194)	0.04	1.640(0.387–6.943)	0.501
TMTV > best cut-off	3.224 (1.156–8.997)	0.025	/	
TLG > median	1.920 (0.687–5.365)	0.213	/	
SUVmax > median	1.479 (0.530–4.128)	0.455	/	
Dmax > median	4.186 (1.452–12.071)	0.008	1.663 (0.110–24.965)	0.712
DmaxVox > median	3.652 (1.291–10.333)	0.015	0.9825(0.077–12.534)	0.989
IMPI intermediate–high	0.755 (0.198–2.884)	0.683	/	

Abbreviations: IPI, International prognostic index; LDH, lactate dehydrogenase; TMTV, total metabolic tumor volume; TLG, total lesion glycolysis; SUVmax, maximum standardized uptake value; Dmax, the distance between the two lesions that are the furthest apart; DmaxVox, the distance between the two voxels that are the furthest apart; IMPI, International Metabolic Prognostic Index; HR, hazard ratio.

**Table 4:** Predictive factors of overall survival.

Variable	HR (95% CI)	*p* Value
Age ≥ 65 years old	1.862 (0.506–6.849)	0.35
Male	1.629 (0.470–5.650)	0.442
Stage III–IV	1.599 (0.458–5.576)	0.461
B symptoms	3.064 (0.540–17.382)	0.206
IPI score 3–5	10.656 (2.542–44.665)	0.001
ECOG ≥ 2	1.72 (0.142–20.845)	0.669
LDH	2.389 (0.687–8.308)	0.171
TMTV > median	2.481 (0.714–8.624)	0.153
TMTV > best cut-off	2.481 (0.714–8.624)	0.153
TLG > median	2.755 (0.785–9.669)	0.114
SUVmax > median	1.172 (0.337–4.082)	0.802
Dmax > median	3.050 (0.856–10.864)	0.085
DmaxVox > median	2.715 (0.774–9.527)	0.119
IMPI intermediate–high	0.552 (0.114–2.672)	0.461

Abbreviations: IPI, International prognostic index; LDH, lactate dehydrogenase; TMTV, total metabolic tumor volume; TLG, total lesion glycolysis; SUVmax, maximum standardized uptake value; Dmax, the distance between the two lesions that are the furthest apart; DmaxVox, the distance between the two voxels that are the furthest apart; IMPI, International Metabolic Prognostic Index; HR, hazard ratio.

**Table 5:** Metabolic parameters according to IMPI score.

	Low IMPI Score (*n* = 43)	Intermediate and High IMPI Score (*n* = 10)	*p*
Median SUVmax	Median SUVmax	18.2	n.s.
Median TMTV	Median TMTV	310.1	0.001
Median TLG	Median TLG	1566.5	n.s.
Median Dmax	Median Dmax	19.9	n.s.
Median DmaxVox	Median DmaxVox	28.4	n.s.

Abbreviations: SUVmax, maximum standardized uptake value; TMTV, total metabolic tumor volume; TLG, total lesion glycolysis; Dmax, the distance between the two lesions that are the furthest apart; DmaxVox, the distance between the two voxels that are the furthest apart; n.s., not significant.

## Data Availability

Data are available upon request to the corresponding author.

## Supplementary Materials

The following supporting information can be downloaded at: https://www.mdpi.com/article/10.3390/curroncol33070392/s1, Table S1: predictive factors of progression-free survival; Table S2: predictive factors of overall survival.

## Abbreviations

The following abbreviations are used in this manuscript:

CAR	Chimeric antigen receptor
CI	Confidence interval
CNS	Central nervous system
CR	Complete response
COO	Cell-of-origin
CTCAE	Common Terminology Criteria for Adverse Events
DLBCL	Diffuse large B-cell lymphoma
Dmax	The distance between the two lesions that are the furthest apart
DmaxVox	The distance between the two outermost voxels
[18F]FDG PET/CT	[18F]-fluorodeoxyglucose positron emission tomography/computed tomography
GCB	Germinal center B-cell-like
HR	Hazard ratio
IHC	Immunohistochemistry
IMPI	International Metabolic Prognostic Index
IPI	International Prognostic Index
LDH	lactate dehydrogenase
NHL	Non-Hodgkin lymphoma
OS	Overall survival
Pola-R-CHP	Polatuzumab-rituximab-CHP
PD	Progressive disease
PS	Performance status
PFS	Progression-free survival
PR	Partial response
RT	Radiation therapy
R-CHOP	Rituximab, cyclophosphamide, doxorubicin, vincristine, prednisone
R-COMP	Rituximab, cyclophosphamide, non-pegylated liposomal doxorubicin, vincristine, prednisone
R-DA-EPOCH	etoposide, prednisone, vincristine, cyclophosphamide, doxorubicin, and rituximab
R-DHAOx	Rituximab, oxaliplatin, high-dose cytarabine and dexamethasone
R-GemOx	Rituximab, gemcitabine and oxaliplatin
R-VNCOP-B	rituximab, cyclophosphamide, mitoxantrone, vincristine, etoposide, bleomycin and prednisone
ROC	Receiver operating characteristic
R/R	Relapsed or refractory
SD	Stable disease
SUVmax	Maximum standardized uptake value
TLG	Total lesion glycolysis
TMTV	Total metabolic tumor volume
VOI	Volume of interest
WHO	World Health Organization
