# Supplementary material for: Prognostic Value of Semi-Quantitative Metabolic Parameters on [18F]FDG PET/CT in Patients with Diffuse Large B-Cell Lymphoma at Diagnosis
Source: Curr Oncol. 2026 Jul 1;33(7):392. doi: 10.3390/curroncol33070392 (PMC13408989; doi:10.3390/curroncol33070392)
Supplement: Supplementary file 1 [file curroncol-33-00392-s001.zip › curroncol-4352860-supplementary.pdf]

Supplementary material

Table S1: predictive factors of progression-free survival

Variable	HR (95% CI)	P value
TMTV > median (n=49)	3.498 (1.211-10.103)	0.02
Dmax > median (n=45)	3.274 (1.088-9.849)	0.034

Table S2: predictive factors of overall survival

Variable	HR (95% CI)	P value
TMTV > median (n=49)	3.037 (0.816-11.297)	0.09
Dmax > median (n=45)	2.273 (0.603-8.57)	0.22
